# Characterization of orthogeriatric care models and association with mortality and health-economic outcomes in patients with hip fractures: a retrospective cohort study from Germany

**DOI:** 10.1186/s12877-026-08021-5

**Published:** 2026-07-20

**Authors:** Theresa Unseld, Kilian Rapp, Hans-Helmut König, Thomas Friess, Dietrich Rothenbacher, Gisela Büchele, Claudia Konnopka

**Affiliations:** 1 Institute of Epidemiology and Medical Biometry, Ulm University, Ulm, Germany; 2 Department of Clinical Gerontology, Robert-Bosch-Hospital, Stuttgart, Germany; 3 Department of Health Economics and Health Services Research, University Medical Center Hamburg-Eppendorf, Hamburg, Germany; 4 AUC - Akademie der Unfallchirurgie GmbH, Munich, Germany; 5 Center for Trauma Research, Ulm University, Ulm, Germany

**Keywords:** Orthogeriatric care, Rehabilitation, Mortality, Costs, Length of stay

## Abstract

**Background:**

Multidisciplinary collaboration between traumatologists and geriatricians has gained international recognition for improving outcomes for older patients after fragility fractures through various models of care delivery. To inform decisions about harmonizing existing models, a deeper understanding of their delivery and impact on patient-relevant and health-economic outcomes is needed.

**Methods:**

We analyzed health insurance claims data from patients aged *≥* 80 years hospitalized with hip fractures between 2014 and 2019 at 121 certified German orthogeriatric centers. We defined hospital-level orthogeriatric care models by the geriatricians’ integration into the surgical wards (geriatric consult service (GCS) vs. integrated care model (ICM)), their weekly patient visit frequency in hospitals with GCS (> 2 (high) vs. 2 (low)), and the network structure between hospitals (single-site institution vs. multisite network cooperation). Outcomes included survival time, direct inpatient medical costs, length of stay (LOS), delivery of early complex geriatric rehabilitation (EGR), and transfer patterns.

**Results:**

The four care models observed in $\:\ge\:5$ hospitals were low-frequency GCS (26 hospitals, 1479 patients), high-frequency GCS (46 hospitals, 3451 patients), hospital networks (42 hospitals, 1832 patients), and ICM (7 hospitals, 457 patients). Covariate-adjusted death hazards revealed the lowest 30-day hazard in ICM hospitals and the lowest 30-to-180-day hazard in hospital networks. Statistically significant differences were found when comparing these hazards with those in low-frequency GCS hospitals, showing respective reductions of 28% and 20%. Although the care models involved different individual treatment paths, such as varying timings and rates of EGR or transfers to external wards or subacute facilities, their overall costs remained similar.

**Conclusions:**

Our findings indicate that health outcomes in orthogeriatric care models depend not only on the availability of geriatricians but also on the extent of their involvement in patient management, whereas mean costs were similar across all models.

**Supplementary Information:**

The online version contains supplementary material available at 10.1186/s12877-026-08021-5.

## Introduction

Orthogeriatric care is an internationally recognized, multidisciplinary treatment concept designed to improve health-related outcomes in patients with fragility fractures by combining trauma surgery and geriatric expertise [1, 2]. Various care delivery models have emerged to address the unique needs of this vulnerable population [3]. In an integrated care model (ICM), the traumatologist and the permanently integrated geriatrician share responsibility for the patients. In alternative models, either the traumatologist or the geriatrician has primary responsibility, with the other regularly visiting the surgical or geriatric ward for consultation. Treatment in a surgical ward may be followed by an early transfer to a geriatric ward, either on the same site or in a different hospital (sequential model). Finally, after the acute stay, and with approval from the health insurer, patients may be transferred to subacute rehabilitation (SR) facilities. These facilities are usually hospital-based rehabilitation units that specialize in restoring physical function, mobility, and independence in daily life. In these units, daily individual or group therapies are held for patients who meet the requirements for this treatment and for whom a rehabilitation unit is available in the region. In Germany, this therapy typically lasts three weeks, and geriatricians and their multidisciplinary teams can be involved in assessing and treating patients at various rates during the SR stay or acute stay [4].

Previous studies have investigated associations between orthogeriatric care models and patient-relevant health outcomes, primarily focusing on the type of geriatricians’ integration at the surgical ward in single hospitals [5–7]. Less is known about the impact of visit frequency and multisite cooperation structures with external wards. In addition, few studies have directly compared different orthogeriatric care models against each other, especially not in larger samples [7]. Hip fractures are a common type of fragility fracture, and their absolute number is increasing in countries facing demographic changes [8, 9]. Fragility fractures pose a high economic burden on an already strained healthcare system [10]. Therefore, an additional focus on economic outcomes is warranted. Furthermore, to explain mechanisms by which care models may affect health and economic outcomes and contextualize eventual differences, a deeper understanding of the sometimes convoluted transfers of patients between wards and rehabilitative facilities, as well as the associated timing and frequency of rehabilitative care, is necessary [11].

Different delivery models of orthogeriatric co-management also exist in Germany. To improve the standard of orthogeriatric co-management in Germany, both the German Trauma Society *(Deutsche Gesellschaft für Unfallchirurgie (DGU))* and the German Geriatric Association *(Bundesverband Geriatrie (BVG))* have developed a certification process. For centers that have been certified as “orthogeriatric centers *(AltersTraumaZentren (ATZs))*”, detailed information is available regarding the specific care delivery model applied, including how and to what extent geriatricians were involved in the treatment of patients with fractures. Thus, our study aimed to compare different models of orthogeriatric care delivery in these well-characterized certified orthogeriatric centers regarding survival time, costs, length of stay, and post-surgical treatment pathways, as observed in nationwide health claims data. We hypothesized that these outcomes would be associated with hospital-level organizational structures relating to the integration of geriatricians into surgical wards, geriatricians’ visiting frequency, or hospital network structure.

## Methods

### Study design and data source

The research questions were analyzed using a retrospective cohort study of claims data. Data were provided by the scientific institute of the Allgemeine Ortskrankenkasse (*Wissenschaftliches Institut der AOK (WIdO)*,* Berlin*,* Germany*). The Allgemeine Ortskrankenkasse (AOK) is the largest statutory health insurance association in Germany, covering approximately one-third of the population [12]. Data on hospital and SR stays and patient characteristics were available from January 1, 2011, through December 31, 2019. To allow for sufficient wash-in and follow-up periods, we restricted the study period to January 1, 2014, through December 31, 2018.

### Study sample

#### Hospitals

The DGU and the BVG provided most of the structural factors defining the orthogeriatric care models. These organizations can certify German hospitals as ATZs [13]. An independent body accredits three-year certification based on compliance with defined standard operating procedures. Certification requires structured orthogeriatric co‑management, including regular joint geriatric and surgical rounds, as well as interdisciplinary care pathways for delirium, osteoporosis, malnutrition, and a level II geriatric assessment according to the guidelines of the Working Group of Scientific Medical Societies (*Arbeitsgemeinschaft der Wissenschaftlichen Medizinischen Fachgesellschaften* (AWMF)) [18, 48]. Apart from the standard operating procedures required for certification, implementation details may vary between hospitals and were not included in the DGU/BVG certification checklist. For hospitals where such implementation details were missing, we requested an anonymous response via email. If there was no response, we followed up with phone calls. Details about the query, including response numbers, can be found in the Supplement.

#### Patients

The target population included patients aged 80 years and older who were admitted to an eligible hospital during the study period (January 1, 2014, to December 31, 2018) and discharged with a hip fracture classified by the International Code of Diseases, tenth revision (ICD-10), as S72.0 or S72.1. In addition to patients with an ICD-10 hip fracture diagnosis at discharge, the study included patients with a hip fracture diagnosis at admission and a pathological osteoporotic fracture diagnosis (ICD-10 code M80) at discharge, which does not include oncological causes. Out of these hospitalization records, we selected the first hospitalization within the study period for each patient as the “index fracture” record. In order to investigate whether certain forms of orthogeriatric co-management offer an advantage, we ensured that the included patients actually required care from geriatricians. In most cases, this is the case starting at age 75. The higher age limit of 80 years was chosen because patients of this age are considered geriatric by definition, due to their increased frailty and related complications, and must be co-managed by geriatricians [14, 15]. We excluded index fractures with recent records of previous fractures, those with implausible SR transfer dates, and those who were already hospitalized for another diagnosis at the time of fracture. Since surgery is the standard procedure for hip fractures, the main statistical analyses were restricted to surgically treated index fractures with operating and procedure code (OPS) codes 5-790, 5-793, or 5-794, combined with the final digit “e” (femoral neck) or “f” (proximal femur), or OPS 5-820 (repositioning of fractures and dislocations, or hip protheses). Details on the exclusion criteria are provided in the Supplement.

### Orthogeriatric care models

We distinguished hospital-level orthogeriatric care models based on the following structural factors in the surgical ward where the patients underwent surgery:

1. Integration of the geriatrician into the surgical ward: integrated geriatric expertise in trauma sur-gery in which geriatric expertise is equally integrated into patient care and decision-making pro-cesses with the geriatrician as a permanent member of the surgical ward (integrated care model, ICM) vs. a geriatric consulting service, in which the geriatrician regularly consults with the surgical ward (GCS)
2. Visiting frequency of the geriatrician to the patients: 2 visits weekly (low, as the minimum required for certification) vs. >2 visits weekly (high)
3. Hospital network structure: single-site institution vs. multisite network cooperation, in which the treating hospital regularly collaborates with other external sites, e.g., by transferring patients.

The first factor relates to how responsibilities are divided between the geriatric and the surgical care teams during the preoperative and postoperative phases on the surgical ward. Traditionally, patients with osteoporotic fractures were treated on surgical wards, with geriatric involvement only upon medical request [3]. Orthogeriatric co-management, however, is a multidisciplinary approach that aims to provide better care for frail patients through more intense professional collaboration [2]. This collaboration can be achieved through regular geriatric visits to the surgical ward (GCS) or in an ICM, in which geriatric expertise is equally integrated into patient care and decision-making processes [5]. In Germany, patients are typically admitted through accident and emergency doctors. Upon admission to the ATZ, specific scores and assessments must be administered —e.g., screening according to the Identification of Seniors at Risk score [16] or Lachs [17], or frailty assessments — to evaluate the risk of delirium and, subsequently, nutritional deficiencies. Comprehensive geriatric assessments are used during the patient’s initial geriatric evaluation, at the latest as part of determining the indication for or initiating complex geriatric care. Nursing staff, physical and occupational therapists, and specially qualified liaison staff are also involved in administering the scores and assessments. The emergency department team usually includes a trauma surgeon. According to the criteria for certification as a geriatric trauma center, geriatric expertise must be available, but it is not mandatory for it to be applied preoperatively [18]. Anesthesiologists are directly involved in the perioperative care but are only integrated into the broader orthogeriatric co-management if a postoperative stay in the intensive care unit is required. The goal is to operate on patients with hip fractures within 24 h [19, 20].

Previous literature suggests that greater geriatric involvement in the acute post-operative period results in better patient outcomes than less frequent involvement [3, 5]. To better understand the impact of the timing and frequency of geriatric involvement, we examined how frequently geriatricians visited patients as a second factor. Certification to an ATZ requires geriatricians to be present on the surgical ward in person at least twice a week, including for bedside visits [18]. Therefore, we classified the visit frequency to patients as 2 or more than 2 visits per week. As a third factor, we considered the hospital network structure, implying multisite cooperation and routine patient transfers across sites. A hospital network composition may work by managing patients’ surgery and initial postoperative phase in surgical wards with geriatric consultation, followed by a transfer to an external geriatric ward within this network for early rehabilitation [3]. Patients in hospital networks are usually transferred to external hospitals for acute geriatric rehabilitation, as described in the next section. These transfers do not include transfers to subacute rehabilitation.

**Fig. 1:**
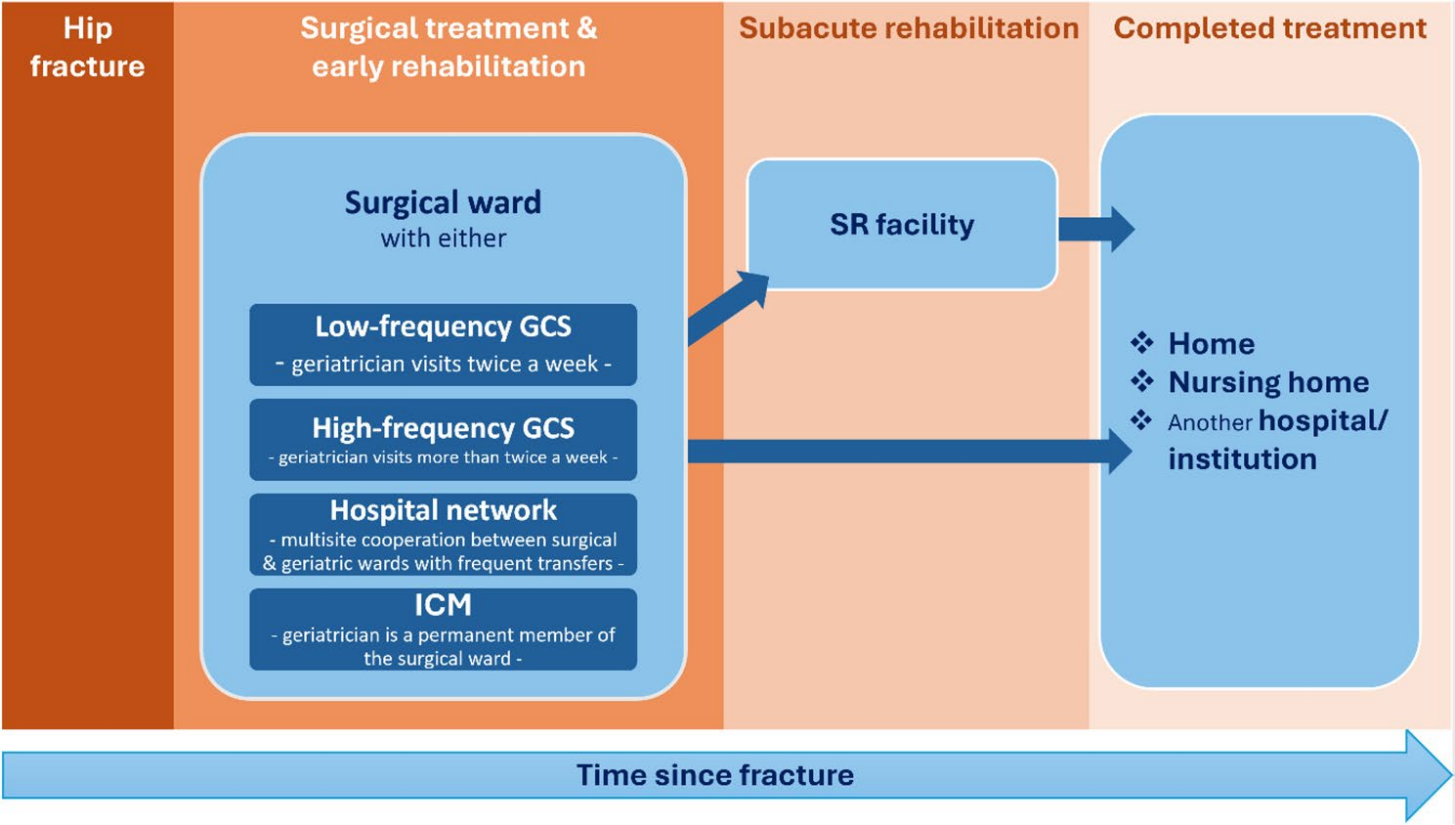
Patient pathways from admission with a hip fracture until treatment completion. ICM: integrated care model with shared patient responsibilities of a trauma surgeon and geriatrician. GCS: geriatric consult service. SR: subacute rehabilitation

Using these factors, we identified combinations occurring in at least five certified hospitals in our data (Table S1). This resulted in the following orthogeriatric care models (Fig. 1):

(A) Single-site GCS with low visit frequency ("low-frequency GCS")
(B) Single-site GCS with high visit frequency ("high-frequency GCS")
(C) Network with GCS, but structures in place for transfers to external geriatric wards for early reha-bilitation ("hospital network")
(D) Single-site ICM ("ICM")

### Hospital characteristics

In addition to the structural factors listed above that were used to define orthogeriatric care models, we investigated hospital characteristics relating to the volumes of the surgical and geriatric wards. A hospital’s surgical volume was defined as the number of yearly treated patients with fragility or hip fractures. A hospital’s geriatric volume was defined as the number of yearly early complex geriatric rehabilitation (EGR) therapies performed. EGR is an intensive, interdisciplinary program initiated shortly after fracture, designed to prevent functional decline and support independence in older patients. It involves 10, 20, or 30 treatment units delivered over 7, 14, or 21 days (OPS codes 8-550.0, 8-550.1, 8-550.2) by physiotherapists, occupational therapists, speech therapists, and specially trained nursing staff, under the joint supervision of geriatricians and trauma surgeons. Eligibility requires structural prerequisites, such as standardized assessments and qualified personnel, as well as patient-specific criteria, including multimorbidity, frailty, or cognitive impairment combined with acute medical needs. EGR thus targets patients with simultaneous curative and rehabilitative requirements, offering a more comprehensive approach than standard surgical trauma care [21]. To account for regional variations in AOK coverage, we weighted the hospital volumes by the inverse of yearly state-level coverage proportions, defined as the number of AOK-insured patients [22] divided by the total population per federal state [23] (*Table S2*).

### Patient characteristics

Regarding sociodemographic characteristics, we considered patients’ sex and age at the time of hospital admission due to an index hip fracture (“index admission”). As covariates associated with patients’ health status, we considered their care levels and a medication-based comorbidity score [24], which is the sum of medication prescriptions for 22 predefined disease groups, within one year prior to the index admission. In Germany, the care level (*Pflegegrad*) is the official classification used to determine the extent of long-term care needs and the corresponding cash allowance or benefits-in-kind provided by the statutory long-term care insurance system [25]. Levels range from 1 (minor impairments of independence) to 5 (most severe impairments with special care requirements) and are based on standardized assessments of mobility, cognitive and communicative abilities, behavioral issues, self-care, and everyday life management [26]. For patients who were already hospitalized at any time during the preceding year, we reported the sum of inpatient costs from the preceding year.

### Spatial characteristics

Previous studies have found that socioeconomic status (SES) is a major determinant of regional health status [27–29]. Since the orthogeriatric care models in Germany historically depended on the federal states [30], regional inequalities may confound the association between orthogeriatric care models and mortality and health-economic outcomes such as survival time and inpatient costs. For example, existing regional infrastructures may influence the hospitals’ choice of orthogeriatric care model. In states where hospitals historically transferred many patients to SR facilities, this type of rehabilitation may still be popular, which could lead to associations of regional SES and survival time or inpatient costs. Furthermore, hospitals in federal states with fewer resources may opt for models that consolidate resources in one place. To account for differences in regional SES, we considered the German Index of Socioeconomic Deprivation (GISD) [31] by federal state. This validated index ranges from 0% (lowest deprivation) to 100% (highest deprivation) and includes indicators of household income, employment, and education.

### Mortality and health-economic outcomes

As main outcomes of interest, we analyzed the time from index admission to death within 180 days of index admission and the mean direct healthcare costs during the index hospital and first SR stay that followed. We focused on inpatient costs because they are the primary driver of costs associated with fragility hip fractures [4, 10, 32] and applied a payer perspective since health insurances reimburse almost all costs for fractures in Germany. These costs could include surgery, rehabilitation therapy, or additional secondary diagnoses. The factors (1)-(3) from above define the orthogeriatric care models and refer to structures of the surgical ward where the patients undergo surgery. However, they do not guarantee particular treatment paths at the patient level. Hence, we were also interested in transfers between hospitals, the timing, length, frequency, and type (EGR and/or SR) of rehabilitative treatment, and the length of stay (LOS) in hospitals and in SR facilities.

### Statistical analysis

For the survival analyses, we estimated nonparametric Kaplan-Meier curves stratified by the orthogeriatric care model and covariate-adjusted death hazard ratios in Cox proportional hazards models. As healthcare costs, we considered the sum of direct healthcare costs reimbursed for the inpatient hospital or SR stays until discharge (“costs in hospital and SR”). Covariate-adjusted cost ratios were estimated in gamma regression models with a logarithmic link function. In the main regression models for the death hazards and costs, we included covariate groups of sex and age (as demographic factors), the patients’ care level and comorbidity score (as indicators of the patients’ health status at baseline), and the GISD summarizing spatially varying SES characteristics in a validated index (as regional factors). All of these covariates were considered as potential confounders according to the modified disjunctive cause criterion [33], given that the orthogeriatric care model was not formally randomized to the patients. Dependencies of patients treated in the same index hospital were accounted for by using robust variance estimation (the grouped jackknife method for the survival analysis or generalized estimating equations for the cost analysis). Besides the total costs in the hospital and SR, we analyzed cost components as inpatient costs per case from index admission to the first hospital discharge (“costs in hospital”) and inpatient costs per case from the first SR admission after the index fracture to the SR discharge (“costs in SR”). In addition, we analyzed subgroups based on whether patients had received EGR at the time of discharge from the hospital. More details about the statistical survival and cost analysis are provided in the Supplement.

We analyzed baseline, inpatient, and hospital characteristics descriptively. Hospital characteristics and orthogeriatric care models were mapped to patient characteristics based on the hospitals where the patients underwent surgery (“index hospital”). In case of a multisite transfer, the patients’ index hospitals may differ from the hospitals where they received the geriatric rehabilitation (“EGR hospital”). To evaluate hospital-level characteristics relating to both surgery and geriatric rehabilitation, we presented separate statistics for index and EGR hospitals. Metric data were presented as medians with interquartile ranges or means with standard deviations (SDs), and categorical data were presented as frequencies with percentages. The spatial variables were mapped to the patients via their year of index admission and their residential federal state.

All statistical analyses were performed using R (version 4.5.0) [34].

### Sensitivity analyses

The sensitivity of the regression results to the covariate adjustments was investigated by considering alternative adjustment sets. This was done by investigating the changes in estimates and confidence intervals when different combinations of covariate groups were excluded. We considered thematically connected covariate groups of the above-described demographic factors, health status, and regional factors and fitted the regression models for all combinations of excluding these covariate groups. We used a directed acyclic graph to explain potential causal pathways between the orthogeriatric care model and the main outcomes, survival time and inpatient costs.

Adverse events may shorten the length of stay. To evaluate how this influences cost estimates, we performed descriptive subgroup analyses, excluding patients who died during their hospital or SR stay and patients who were transferred back to the hospital from the SR facility. Since patients may be discharged home and subsequently die, we also investigated patients who died within 21 days of starting the SR stay, in addition to those who died during the SR stay. We considered this to less likely occur during the acute stay since patients would likely be kept in the hospital. Twenty-one days is a common length of an SR stay in Germany and was also the median LOS in SR for patients discharged alive from SR in our study. In the following sections, the term “patients discharged alive” refers to those who did not die in the hospital or during SR or were transferred back to the hospital from SR. Further specifications will be made if specific subgroups are addressed, e.g., “patients discharged alive from hospital.”

## Results

### Description of the study population and the included hospitals

Across all eligible hospitals, ICM was implemented less frequently than any form of GCS (Table 1). While the age distribution was similar across hospitals, the proportions of females and of patients without a care level or with care level one were slightly higher in ICM hospitals than in hospitals with the other orthogeriatric care models. For all models, approximately 40% of patients were hospitalized in the year before admission, with similar inpatient costs. Most of the patients received surgical treatment within one day of index admission, with the highest percentage in ICM hospitals. Almost all patients received surgical treatment and less than 1.5% died without surgery across all orthogeriatric care models (*Table S3*). Among hospitals where patients underwent surgery, the average volume of fragility fractures was highest among those with ICM, while the average volume of EGR was highest among those with high-frequency GCS. However, among hospitals where patients received EGR, including external wards to which patients may be transferred, the average volume of EGR was highest among hospital networks. The timeline of hospital numbers and hospital-level characteristics over the follow-up years in *Table S4* showed the strongest increase in hospital numbers for hospital networks and high-frequency GCS. Except for ICM, the index hospitals’ volumes of yearly hip fractures were increasing, with the strongest increase for high-frequency GCS. The hospitals’ yearly volume of EGR showed some variations over the follow-up years but remained consistently highest in hospital networks. Regarding socioeconomic characteristics, Table 1 shows that patients treated in ICM hospitals were from federal states with higher socioeconomic deprivation, and patients treated in high-frequency GCS hospitals were from federal states with lower socioeconomic deprivation than patients treated in hospitals with the other orthogeriatric care models.

**Table 1:** Baseline characteristics by the index hospitals’ orthogeriatric care model

Characteristic	low-frequency GCS^1^	high-frequency GCS^1^	hospital network^1^	ICM^1^
	1479 patients	3451 patients	1832 patients	457 patients
	26 hospitals	46 hospitals	42 hospitals	7 hospitals
Age at admission (years)	87 (84, 91)	87 (84, 91)	87 (83, 91)	87 (84, 91)
Sex female	1,136 (77%)	2,606 (76%)	1,393 (76%)	376 (82%)
Care level				
None or 1	485 (33%)	1,185 (34%)	601 (33%)	197 (43%)
2–3	648 (44%)	1,416 (41%)	832 (45%)	182 (40%)
4–5	346 (23%)	850 (25%)	399 (22%)	78 (17%)
Resident of a nursing home	363 (25%)	844 (24%)	485 (26%)	97 (21%)
Medication-based comorbidity score	4 (3, 5)	4 (3, 5)	4 (3, 6)	4 (2, 5)
Inpatient costs in the previous year				
No costs in previous year	839 (57%)	1,966 (57%)	1,058 (58%)	281 (61%)
< 5 thsnd. €	336 (23%)	868 (25%)	445 (24%)	105 (23%)
≥ 5 thsnd. €	304 (21%)	617 (18%)	329 (18%)	71 (16%)
Surgery within 1 day of admission	1,275 (86%)	2,902 (84%)	1,549 (85%)	407 (89%)
Year of index admission				
2014	0 (0%)	65 (1.9%)	58 (3.2%)	0 (0%)
2015	205 (14%)	399 (12%)	88 (4.8%)	32 (7.0%)
2016	285 (19%)	692 (20%)	224 (12%)	105 (23%)
2017	435 (29%)	994 (29%)	606 (33%)	138 (30%)
2018	554 (37%)	1,301 (38%)	856 (47%)	182 (40%)
Hospital volume where patients underwent surgery:				
Yearly treated hip fractures	419 (308, 503)	415 (302, 565)	406 (309, 500)	523 (306, 650)
Yearly EGR	244 (167, 356)	285 (206, 504)	0 (0, 186)	372 (72, 395)
Hospital volume where patients received EGR:				
Yearly treated fragility fractures	397 (278, 531)	406 (296, 544)	337 (261, 440)	646 (306, 650)
Yearly EGR	338 (222, 416)	303 (212, 511)	501 (337, 693)	372 (112, 395)
Socioeconomic factors in patients’ residential state:				
German Index of Socioeconomic Deprivation (%)	67 (38, 68)	36 (35, 65)	46 (38, 67)	100 (36, 100)

^1^*n* (%) or median (1st quartile, 3rd quartile), *thsnd* thousand, *EGR* early complex geriatric rehabilitation therapy, *GCS* geriatric consulting service, *ICM* integrated care model

### Description of mortality and health-economic outcomes and transfer characteristics

**Fig. 2:**
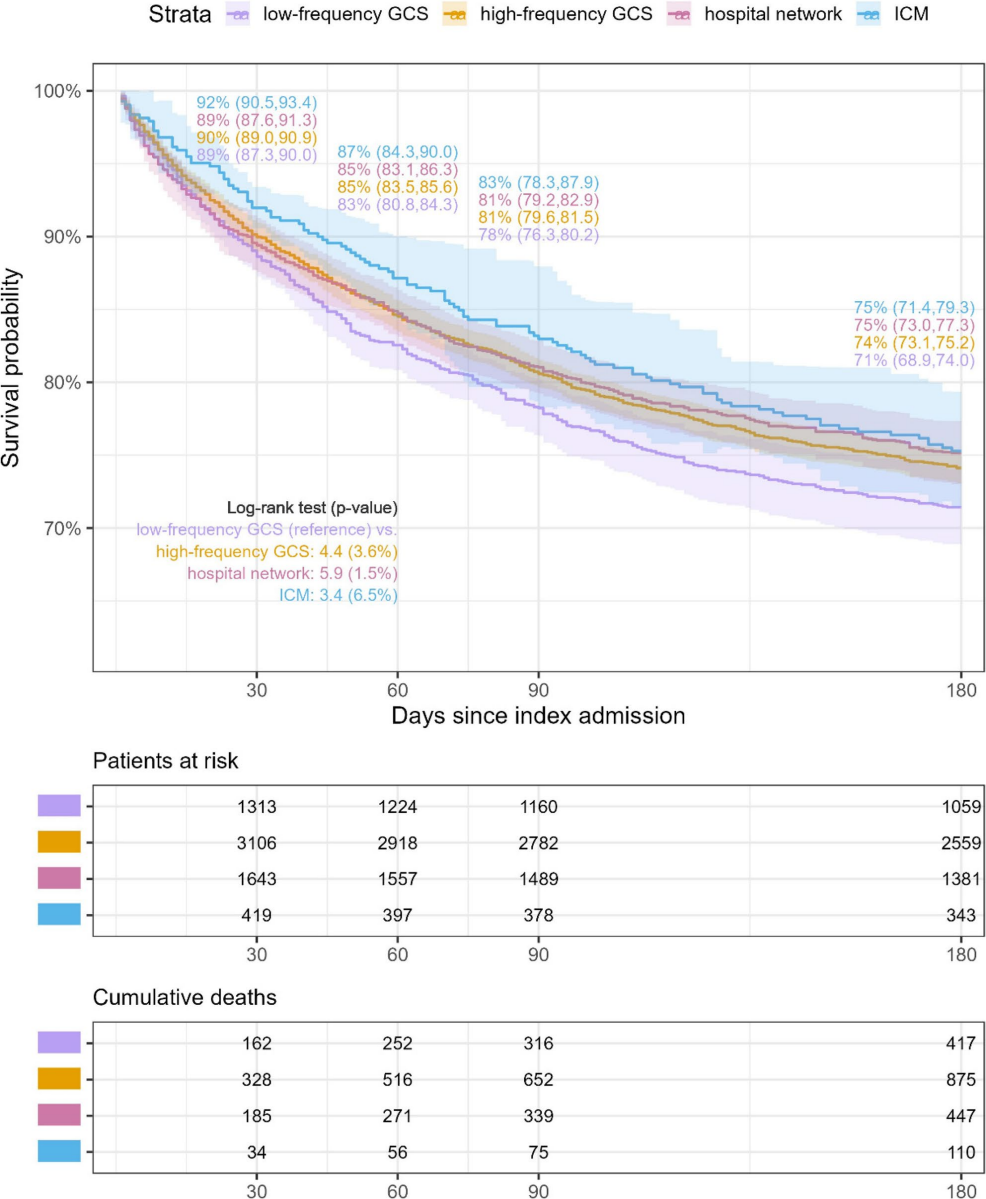
Kaplan–Meier plot of survival probabilities stratified by the orthogeriatric care model and log-rank tests comparing low-frequency GCS (as reference with the lowest level of geriatric involvement) against the other care models. GCS: geriatric consulting service. ICM: integrated care model

Kaplan-Meier estimates revealed the lowest 180-day mortality (or highest survival probabilities) for ICM and hospital networks (Fig. 2). In contrast, low-frequency GCS exhibited the highest mortality, approximately 4% higher. Throughout the 180-day follow-up period, the survival curves flattened, reflecting a decline in death hazards. This decline was most prominent in hospital networks, where survival trajectories gradually approached those of ICM.

Mean costs of the combined stay in hospital and SR were similar across the orthogeriatric care models (Table 2). Larger differences emerged when neglecting the effect of extreme costs by considering medians instead of means (*Table S5*): due to lower costs during the hospital stay correlated with slightly shorter hospital LOS, the median costs of combined hospital and SR stays were lower in ICMs, especially when compared to low-frequency GCS and hospital networks.

**Table 2:** Treatment and transfer characteristics by the hospitals’ orthogeriatric care model

Characteristic	low-frequency GCS^1^	high-frequency GCS^1^	hospital network^1^	ICM^1^
Costs in hospital and SR (thsnd. €)	12.3 (8.3)	12.5 (6.1)	12.1 (5.1)	12.2 (7.7)
LOS in hospital and SR	24 (14, 35)	26 (16, 37)	28 (16, 36)	23 (17, 35)
(Length of) EGR*				
No EGR	773 (52%)	1,341 (39%)	858 (47%)	206 (45%)
7 days	61 (4.1%)	200 (5.8%)	68 (3.7%)	19 (4.2%)
14 days	577 (39%)	1,714 (50%)	714 (39%)	180 (39%)
21 days	68 (4.6%)	196 (5.7%)	192 (10%)	52 (11%)
Days to EGR start	7 (4, 10)	3 (1, 6)	10 (7, 13)	2 (2, 4)
EGR during 1st stay†	657 (93%)	2,070 (98%)	299 (31%)	238 (95%)
Transfer to SR*	420 (31%)	1,250 (39%)	413 (25%)	149 (35%)
Days until transfer to SR	21 (15, 29)	18 (15, 24)	18 (12, 26)	17 (14, 24)
Transfer to an external hospital*	81 (5.5%)	71 (2.1%)	767 (42%)	20 (4.4%)
Days until 1st transfer	11 (9, 15)	11 (8, 15)	11 (9, 13)	13 (9, 17)
LOS in transferred hospital	20 (11, 23)	19 (13, 23)	19 (15, 22)	16 (14, 21)
EGR in transferred hospital‡	46 (59%)	38 (58%)	651 (89%)	13 (65%)
≥ 2 transfers‡	11 (14%)	11 (15%)	46 (6.0%)	4 (20%)
Volume of hospital transferred to‡				
Yearly treated fragility fractures	238 (201, 378)	216 (100, 446)	323 (237, 435)	225 (142, 320)
Yearly EGR	253 (0, 468)	135 (0, 285)	501 (314, 845)	246 (77, 289)

^1^*n* (%), mean (standard deviation) or median (1st quartile, 3rd quartile), *thsnd* thousand, *GCS* geriatric consulting service, *ICM* integrated care model, *EGR* early complex geriatric rehabilitation therapy, *SR* subacute rehabilitation, *LOS* length of stay.

Percentages are computed in all patients or in subgroups of patients discharged alive from hospital (*), patients with EGR (†), or transferred patients (‡)

EGR and SR were the main drivers of LOS (Figure S1). Across all orthogeriatric care models, patients without EGR had shorter hospital LOS since EGR required a minimum inpatient treatment duration of seven days. Variations in LOS and costs of the combined hospital and SR stay in this group then mainly depended on whether patients were transferred to SR. Among patients with EGR, those treated in hospitals with ICM and high-frequency GCS had shorter LOS than those treated in hospitals with low-frequency GCS, and even shorter LOS than those treated in hospital networks (Table 2, Figure S1). This correlates with the earlier start of EGR in the former hospitals.

Among hospital networks, 69% of EGR occurred only after transfer to an external ward, whereas 93–98% of patients in single-site models received EGR in the hospital where they received surgical treatment. The higher proportions of transfers to external wards in hospital networks than in the other orthogeriatric care models were accompanied by lower proportions of transfers to SR facilities, thereby comprising the SR component in the combined hospital and SR costs. The transferred patients in hospital networks were transferred to hospitals with higher geriatric volumes, where they were more likely to receive EGR and less likely to undergo multiple transfers than patients transferred from hospitals with the other models.

An investigation of adverse events that may shorten LOS and affect costs showed that 7–9% of patients who underwent surgery died in the hospital afterwards, but median hospital costs remained similar when excluding these patients (Table S5). However, excluding these patients, as well as those who died during SR or who were transferred back to the hospital from SR, increased the combined hospital and SR stay costs with largest increases for high-frequency GCS hospitals. This was due to higher SR rates in these patients than in the overall population, since patients who die in hospital cannot incur subsequent costs in SR. Readmissions were the most common adverse event that could lead to an increase in death rates or costs later on. Readmissions occurred in 6–7% fewer patients discharged from hospital networks or ICM hospitals than in those discharged from low- or high-frequency GCS hospitals.

### Covariate-adjusted comparisons of death hazards and inpatient costs

The above-described Kaplan-Meier curves indicated non-proportionality of the death hazards in a non-parametric model without covariates. To investigate whether non-proportionality remained after covariate adjusting, we investigated Schoenfeld residual plots and tests (Figure S2). These diagnostics indicated non-constant hazard ratios for comparisons that included the hazards in ICM hospitals or hospital networks: early on, ICM had a large protective association compared to low-frequency GCS or hospital networks, but this risk reduction waned over time and turned into a neutral or slightly increased hazard ratio. In contrast, hospital networks had a higher early hazard than low-frequency GCS but a constantly lower hazard for later follow-up times when most of the transferred patients in hospital networks had received the EGR at the external ward. To illustrate these trends, we fitted hazards over two different time intervals. We chose 30 days as a cut point since the Schoenfeld plots indicated that it can separate the aforementioned early associations of treatment in ICM hospitals or hospital networks with the death hazard from later associations. The resulting estimates showed the lowest 30‑day hazard in ICM hospitals and, thereafter, the lowest hazards in hospital networks (Table 3). The corresponding 30-day HR for ICM and 30-180-day HR for hospital networks were significant when compared to low‑frequency GCS and adjusting for multiple pairwise testing. Treatment in high-frequency GCS hospitals was associated with a rather constant 11–12% hazard reduction over low-frequency GCS and was statistically significant when considering the overall follow-up time without adjusting the confidence intervals for multiple testing (Figure S2). The comparisons of high-frequency GCS with hospital networks and ICM were statistically non-significant, and the time-dependent HRs showed, at a smaller magnitude, the same trends that had been observed in the comparison of these orthogeriatric care models with low-frequency GCS.

**Table 3:** Hazard ratio of death and ratio of mean costs between orthogeriatric care models

Pairwise comparisons	Hazard ratio¹ (0,30] days	Hazard ratio¹ (30,180] days	Cost ratio in hospital & SR¹
Reference: low-frequency GCS			
high-frequency GCS	0.88 (0.72,1.07)	0.89 (0.74,1.06)	1.00 (0.97,1.04)
hospital network	0.92 (0.69,1.23)	0.80 (0.64,0.99)	0.99 (0.95,1.03)
ICM	0.72 (0.56,0.92)	0.99 (0.75,1.32)	1.00 (0.94,1.06)
Reference: high-frequency GCS			
hospital network	1.05 (0.80,1.38)	0.90 (0.74,1.10)	0.98 (0.96,1.01)
ICM	0.82 (0.64,1.04)	1.12 (0.84,1.51)	0.99 (0.94,1.05)
Reference: hospital network			
ICM	0.78 (0.57,1.07)	1.25 (0.92,1.70)	1.01 (0.95,1.07)

*GCS* geriatric consulting service, *ICM* integrated care model

^1^adjusted for age, sex, medication-based comorbidity score, care level, socioeconomic deprivation, within-hospital dependencies, and multiple testing with 95% confidence intervals

Sensitivity analyses supported the robustness of the hazard comparisons for high‑frequency GCS and hospital networks versus low‑frequency GCS across covariate adjustment sets (Table S6). Confidence interval widths were similar between the adjustment sets, and absolute changes in estimates were ≤ 0.05. The HR of ICM over low-frequency GCS was more sensitive to covariate choice: adjusting for socioeconomic deprivation lowered the HR to 0.64 (30‑day) and 0.89 (30‑to‑180‑day), whereas adjusting for health status attenuated the HRs. Adjusting for age and sex had smaller effects on the estimated HRs. The inclusion of non‑operated fractures in an analysis without left truncation reduced differences in 30‑day hazards and widened confidence intervals (Table S7), while 30‑to‑180‑day hazards were less affected.

Regarding the costs of combined hospital and SR stay, multiplicity- and covariate-adjusted comparisons revealed no statistically significant differences between the orthogeriatric care models. Cost ratios remained close to one across all adjustment sets, with ratios relative to low‑frequency GCS ranging from 0.98 to 1.02 (Table S6). When considering only the costs of the hospital stay in patients with EGR, however, a reduction of 5% was estimated for those treated in hospitals with high-frequency GCS or ICMs over those treated in hospitals with low-frequency GCS and a reduction of 8% over those treated in hospital networks (Figure S3). Likewise, a shorter covariate-adjusted hospital LOS in the former patients was observed, mirroring the descriptive results from the previous section, with statistically significant reductions of 10% and 19% over those treated in hospitals with low-frequency GCS and 22% and 30% over those treated in hospital networks.

## Discussion

### Summary and discussion of the results

This nationwide cohort study compared four orthogeriatric care models in Germany regarding mortality and health-economic outcomes after hip fracture in older patients. The lower mortality observed in hospitals that implemented hospital networks, single-site ICM, or high-frequency GCS, compared to those that employed low-frequency GCS, indicates that the intensity and consistency of geriatric care play pivotal roles in achieving optimal patient outcomes, particularly during EGR. Hospitals where the geriatrician was integrated in the surgical ward (ICM) showed the lowest covariate-adjusted 30‑day death hazard. This likely reflects the acute benefits of early surgery combined with immediate, continuous geriatric involvement, allowing for a multidisciplinary management of pain, intraoperative hypotension, and delirium and an early start of intensive geriatric rehabilitation (EGR). In fact, several before-after studies contrasting ICM with GCS at single hospitals similarly reported improved key care processes and fewer complications during implementation of ICM than GCS, like shorter time to surgery, less delirium, early mobilization, and reduced 30-day mortality [35–37]. Reduction of surgical complications and promotion of early rehabilitation for patients in need have been reported as essential steps to prevent functional loss and associated increased mortality in patients after hip fractures [7, 38]. Since patients treated in hospital networks predominantly received low-frequency GCS during the acute phase at the surgical ward, the benefits of hospital networks over low-frequency GCS came to play only after transfer to an external ward with high geriatric volumes, where the majority of patients received EGR. The hazard reduction over low-frequency GCS was then weaker than the acute hazard reduction in ICM hospitals but lasted for a longer time. Despite delaying the initiation of EGR compared to low-frequency GCS hospitals, the treatment in geriatric wards may allow for an enhanced focus on the patients’ functional recovery and on underlying causes of weakness, including comorbidities [39], e.g., through a greater daily therapy intensity. In addition, they may benefit from better discharge arrangements that ensure continuity of rehabilitation by community physiotherapists or medication prescriptions [38].

A systematic review and random-effects meta-analysis of 37 studies on economic and health-related outcomes in patients with hip fractures found a larger reduction in in-hospital and 1-year mortality of ICM versus non-orthogeriatric standard care than in GCS over standard care [7]. However, the authors concluded that the existing evidence base does not permit formulating a compelling recommendation regarding the optimal orthogeriatric care model. Additionally, the meta-analytic estimates for many patient outcomes were highly heterogeneous, and studies who directly compared orthogeriatric care models against each other instead of against standard care were almost completely lacking. In our study, the lack of significance in the association between patient outcomes and the role of the geriatrician (ICM vs. GCS) when no distinction is made between GCS subcategories at the surgical ward is consistent with these findings. The permanent integration of the geriatrician into the surgical ward in ICM hospitals, or the direct, ward-based geriatric care for selected patients in hospital networks, are likely more effective than infrequent consultation only [40]. Compared to other studies, the in-hospital mortality rate of 7–9% observed in our study may seem high. However, this high value may be due to the fact that our study sample was limited to patients over 80 years of age, for whom mortality is higher than in the overall hip fracture patient population. Second, particularly when EGR is administered, the length of hospital stay is comparatively long, so there is more time for hospital deaths to occur, whereas in cases of earlier discharge, subsequent deaths are not considered hospital mortality.

A Belgian hospital network registry study compared orthogeriatric care models based on the location of admission (surgical vs. geriatric ward), the main responsibility (consultation service vs. integrated care), and, within the consultation service models, the frequency of visits (systematic consultation vs. consultation on request) [41]. Regarding survival, the researchers found benefits for patients treated on geriatric wards with a surgical consultation service but also higher costs. In our study, none of the eligible hospitals admitted patients to geriatric wards. However, parts of the higher survival at higher treatment costs on geriatric wards that were found in the Belgian study may be reflected in patients who received EGR after transfer in our studies’ hospital networks. In contrast, the slightly lower LOS and costs among patients receiving EGR in ICM and high‑frequency GCS hospitals in our study likely reflect the earlier initiation of EGR enabled by the frequent and timely availability of geriatricians after surgery [7, 41]. A geriatrician must initiate EGR. If a geriatrician is not on site daily, as is the case with low-frequency GCS hospitals, the start of EGR is delayed. Additionally, EGR requires comprehensive staffing structures. These structures are only fully in place where geriatricians have full or shared responsibility. Therefore, EGR can be quickly initiated in an integrated care model, but it may be delayed after transfer to a hospital network’s geriatric ward. Nevertheless, compared to complete treatment on a geriatric ward, the hospital networks’ sequential approach might still have lower costs, e.g., by reducing emergency department visits during the initial days on the surgical ward [41].

Besides patient characteristics, the individual costs observed in our study depended mostly on whether and how patients received rehabilitative treatment, either as EGR during the acute stay or as regular SR. In hospital networks, the sequential approach of geriatric rehabilitation in external wards resulted in 600€ higher costs on average for patients receiving EGR than for patients receiving EGR in hospitals with other care models. However, fewer patients with acute EGR in hospital networks received rehabilitation at subacute facilities than those patients who received EGR in hospitals with the other care models. Thus, hospital networks offset the higher costs of treating patients with EGR by transferring fewer of them to SR, thereby reducing the cost associated with SR, which was 4,000 € per case on average. Meanwhile, patients without acute EGR but with SR were discharged early from hospital networks, thereby reducing the hospital LOS and associated costs compared to the other care models. Furthermore, low-frequency GCS performed less EGR and thereby reduced the elevated costs associated with longer hospital LOS due to the later initiation of EGR compared to high-frequency GCS or ICM. Consequently, despite the differences in in‑hospital LOS and in the timing and frequency of EGR and SR, the mean costs of the combined hospital and SR stay were similar across the models.

EGR is a treatment element available in orthogeriatric care. Although the proportion of patients receiving EGR varied across orthogeriatric models, ATZ certification required the availability of orthogeriatric co-management for all analyzed geriatric trauma patients, regardless of whether they received EGR. While the feasibility of EGR for specific patient profiles may be debatable, the age and sex distributions were similar between the orthogeriatric care models, except for slightly larger proportions of females in hospitals with ICM. Furthermore, the results of higher mortality at similar costs compared to the other orthogeriatric care models remained consistent after adjusting for patient characteristics and socioeconomic deprivation. These observations render the hypothesis that, in particular, patients in low-frequency GCS hospitals are too frail or not frail enough for treatment consideration less plausible. An alternative explanation for the low EGR documentation rate may lie in reimbursement mechanics: if treatment is interrupted or discontinued due to adverse events, it cannot be billed and thus remains unrecorded. However, this would again support the other orthogeriatric care models, since interruptions or discontinuations of treatment are generally considered undesirable events that may be attributed, at least in part, to aspects of treatment after admission, given similar patient characteristics before admission.

Although the certification implies standard care procedures in all analyzed hospitals, regional differences in the frequency of transfers to external hospitals or to SR may be due to different historical policies promoting these models [30]. Patients treated in ICM hospitals lived in federal states with higher socioeconomic deprivation compared to states with other care models. This fact may impact patients’ baseline covariates and preoperative health. Additionally, it may also impact the availability of qualified hospital staff, the quality of the long-term rehabilitation, and the effectiveness of discharge processes by community healthcare structures. Regions with higher socioeconomic status are likely more attractive to healthcare professionals, offering better infrastructure, career opportunities, and higher salaries [42, 43]. Adjusting for the socioeconomic deprivation of patients’ residential states strengthened the apparent benefit of ICM, whereas adjusting for comorbidity, especially care level, attenuated the advantage of ICM. While the former variables account for health disadvantages linked to lower SES [44], the latter variables may serve as downstream colliders reflecting regional disparities in healthcare utilization patterns. For example, some federal states more often use formal benefits-in-kind, while older adults in other states generally rely more on informal family care [45]. This could result in a lower representation of higher care levels in states where most patients treated in ICM hospitals lived. Additionally, when comparing patients from states with lower SES to patients of the same age in states with higher SES, the former group may actually have a poorer health status, leading to earlier death. This could contribute to the underrepresentation of older male patients in those states, as they have already died.

Finally, integrating geriatric expertise directly into surgical wards can improve early outcomes but may also increase workforce demands. Therefore, recommendations have been made to strengthen multidisciplinary collaboration by improving communication and streamlining workflows to avoid overburdening staff [41]. Conversely, hospital networks illustrate how coordinated collaboration across wards and institutions can support longer‑term recovery.

### Strengths and limitations

This nationwide study provides an informative and representative analysis of orthogeriatric care models for older patients with surgically treated hip fractures in certified hospitals. Its nationwide design, large sample size, and nearly complete coverage of patients insured by AOK — Germany’s largest association of statutory health insurances — enhance the generalizability of the results to the German population. However, the generalizability of the findings may be limited by the fact that data is from a single provider, even though patients in Germany are free to choose their health insurance provider. AOK policyholders, in particular, tend to have a lower socioeconomic status than policyholders of other statutory or private health insurance providers [46]. Nevertheless, we controlled for GISD and other influencing factors, such as age and gender, and adjusted for differences in the composition of the care models. Therefore, we expect the results of the care model comparisons to be applicable to patients insured by other providers, although absolute costs and mortality rates may differ from those of the German population as a whole. Furthermore, we adjusted for differences in regional insurance coverage in the definition of hospital volumes by weighting them inversely by yearly state-level coverage proportions.

The findings are also relevant beyond the German context because orthogeriatric care models are being adopted in various international healthcare systems [7]. Although the data is from 2014 to 2019 — a period during which an increasing number of centers were certified and various forms of orthogeriatric co-management were developed — these forms have not changed since then and remain consistent with those described in the current international literature. While implementation details may vary in other countries, key characteristics like the integration of the geriatrician in the surgical ward, their patient visit frequency, and hospital network structures are internationally transferable. Hence, a key strength of this study is its detailed differentiation of orthogeriatric care based on these characteristics. While prior research has primarily compared geriatric GCS with integrated care ICM in a binary fashion, we adopted a more nuanced comparison and completed our analysis by investigating treatment pathways and transfer dynamics that help when comparing orthogeriatric care models internationally and explaining differences in associated patient outcomes. Instead of including patients based on the type of rehabilitation received, we defined the orthogeriatric care models at the hospital level, capturing institutional characteristics that shape care delivery in a natural experiment [30]. The statutory health insurance associations stipulate that patients must be transported via the shortest route between their location and the nearest suitable treatment facility [47]. Therefore, baseline patient characteristics in this hospital-level analysis are more similar than in patient-level analyses, unless regional differences affect the availability of these models or patient health outcomes. We acknowledge that patient trajectories within hospitals may vary depending on ward-level capacities [37], network affiliations, and patient-specific factors, such as frailty and postoperative mobility, which determine the applicability and effectiveness of acute and subacute rehabilitative treatment concepts.

All of the hospitals included in this study are certified orthogeriatric centers that are required to perform standardized geriatric assessments according to AWMF guidelines [18, 48]. Additionally, ATZ certification criteria mandate appropriate emergency treatment protocols and daily access to geriatric expertise when urgent perioperative needs arise. These certifications strengthen the consistency of geriatric assessments and guarantee geriatric involvement across centers, even though results of ambulatory or frailty assessments were not available in our dataset. Furthermore, the fact that over 80% of patients in our dataset underwent surgery within one day of admission demonstrates that the surgical pathway is functioning well and that orthogeriatric care is widely used in certified German orthogeriatric centers.

Despite investigating multiple covariate adjustments, residual confounding factors, such as unmeasured frailty, cognitive status, functional decline, care-seeking behavior, and informal care support, may influence both care model assignment and outcomes. Using a directed acyclic graph and sensitivity analyses helped to clarify potential limitations, but it cannot eliminate them. In particular, the role of the socioeconomic situation of the region in which patients live, including social cohesion and attractiveness to healthcare professionals, merits much more discussion, as data on these relations are intricate and details were not investigated in this study. This is particularly relevant in areas where patients are more susceptible, have multiple comorbidities, and lack access to support services [49–51]. Furthermore, it is conceivable that mortality and cost predictions for individual patients could be improved by calculating digital frailty indices. For instance, previous studies have approximated hospital frailty scores and claims-based frailty using international outpatient and inpatient disease classifications derived from hospital and routine data collected during a patient’s hospital stay or within a certain timeframe [52–54]. However, it was not possible to adequately calculate such indices using the routine data available to us, which consisted of data from a German health insurance company and did not include any hospital documentation or functional parameters. Conversely, it is possible to link claims and long-term care data. Long-term care insurance includes a classification into a care level, which describes the extent of the need for care and represents a measure of functional impairment. In addition to the care level, we adjusted for other known correlates of frailty, such as age, sex, socioeconomic deprivation, and medication-based comorbidity score. Although this comorbidity score was developed to quantify the prevalence of chronic diseases in patients, it can inform population health by reflecting multiple disease burdens. Extending these covariates with a digital frailty index may improve prediction, but may also introduce bias in comparative analyses. Frailty indices derived from diagnoses recorded during the index stay can be affected by early death, timing and type of transfer patterns, or differences in orthogeriatric care models, which influence the accuracy and range of diagnoses used to compute such indices. Additionally, regional variation in care‑seeking behavior may lead to a systematic underestimation of frailty in claims data, which could bias adjusted comparisons when orthogeriatric care models differ across regions [54, 55].

Moreover, we note that caution is warranted when interpreting associations in patient subgroups conditional on post-surgical events. In particular, restricting analyses to patients who were discharged alive, without premature rehabilitation discharge, or with EGR may elucidate differences in the costs between comprehensively treated patients and those with adversely shortened stays. However, associations in these subgroups should not be interpreted in isolation. Additionally, while other endpoints, such as long-term care or nursing homes, may be of interest, analyzing these outcomes would require adapting the study population to exclude patients already living in a nursing home at the time of the fracture. In other words, it would involve a different study sample. Finally, temporal changes in the number of patients and hospitals over the study period may confound comparisons if newer adopters differ systematically from early implementers. While trends were delineated, formal modeling was not undertaken due to the insufficient number of hospital cases for statistical estimation when additionally accounting for within-hospital dependencies.

## Conclusions

Among the analyzed orthogeriatric care models, patients treated in hospitals with ICM or in hospital networks had the lowest 180-day mortality, with similar values for high-frequency GCS. ICM and hospital networks offer the most intensive geriatric care among the analyzed models. However, in the case of hospital networks, intensive geriatric care only begins after transfer, so the reduction in the death hazard over low-frequency GCS started later on. Based on our results, common comparisons of orthogeriatric care models based solely on the integration of the geriatrician into the surgical ward are too general. A more nuanced differentiation of orthogeriatric care models suggests that patient outcomes also depend on visit frequency in GCS models and on hospital network structure, which determines the location of and responsibilities during postoperative geriatric treatment for eligible patients.

## Supplementary Information

Supplementary Material 1. “Supporting information for characterization of orthogeriatric care models and association with mortality and health-economic outcomes in patients with hip fractures: a retrospective cohort study from Germany” provid-ing details on the query of structural factors defining the orthogeriatric care model, exclusion criteria, statisti-cal analysis, and supplementary tables (Tables S1–S8) and figures (Figures S1–S5).

## Data Availability

The data analyzed during the current study cannot be shared because they originated from the scientific institute of the AOK ( *Wissenschaftliches Institut der AOK* (WIdO)) and the use is contractually bound.

## Abbreviations

AOK: Allgemeine Ortskrankenkasse
ATZ: certified orthogeriatric center (*AltersTraumaZentrum*)
AUC: Academy of Trauma Surgery (*Akademie der Unfallchirurgie*)
AWMF: Working Group of Scientific Medical Societies (*Arbeitsgemeinschaft der Wissenschaftlichen Medizinischen Fach-gesellschaften*)
BVG: German Geriatric Association (*Bundesverband Geriatrie*)
CI: Confidence interval
DGU: German Trauma Society (*Deutsche Gesellschaft für Unfallchirurgie*)
EGR: Early complex geriatric rehabilitation therapy
GCS: Geriatric consult service
GISD: German index of socioeconomic deprivation
HR: Hazard ratio
ICD-10: International code of diseases, tenth revision
ICM: Integrated care model
LOS: Length of stay
OPS: Operating and procedure codes
SD: Standard deviation
SES: Socioeconomic status
SR: Subacute rehabilitation
