# Supplementary material for: Characterization of orthogeriatric care models and association with mortality and health-economic outcomes in patients with hip fractures: a retrospective cohort study from Germany
Source: BMC Geriatr. 2026 Jul 20;26:968. doi: 10.1186/s12877-026-08021-5 (PMC13390263; doi:10.1186/s12877-026-08021-5)
Supplement: Supplementary file 1 — Supplementary Material 1. “Supporting information for characterization of orthogeriatric care models and association with mortality and health-economic outcomes in patients with hip fractures: a retrospective cohort study from Germany” provid-ing details on the query of structural factors defining the orthogeriatric care model, exclusion criteria, statisti-cal analysis, and supplementary tables (Tables S1–S8) and figures (Figures S1–S5). [file 12877_2026_8021_MOESM1_ESM.pdf]

Supporting information for characterization of orthogeriatric care models and association with mortality and health-economic outcomes in patients with hip fractures: a retrospective cohort study from Germany

Query of structural factors defining the orthogeriatric care model

Information on certification, including certification dates, hospital network structures, and geriatrician responsibilities and visiting frequency, was gathered from the DGU trauma network directory of the Academy of Trauma Surgery (*Akademie der Unfallchirurgie* (AUC)) and the German Geriatric Association (*Bundesverband Geriatrie*). For 31 hospitals, we inquired about their geriatricians' roles and visit frequencies by contacting the hospitals directly. We sent a standardized email to the contact persons, informing them of the study's purpose and asking for the missing information. Hospitals could respond directly to our institute director (DR) via email, fax, or phone. If there was no response, a reminder email was sent to the hospitals in December 2020. If there was still no response, DR contacted the hospitals by phone calls until January 2021. The final list contained 169 certified hospitals, 124 of which had surgically treated patients aged ≥80 years with hip fractures during the study period. The scientific institute of the AOK combined this information with the patient data based on the institute's indicators and provided us with an anonymized dataset. The institute indicator uniquely identifies the hospitals for reimbursements of medical and rehabilitative services by Social Security providers and was obtained from a reference database of machine-readable quality reports from the Joint Federal Committee.

Details on the exclusion criteria

We excluded hip fractures that occurred within four years of a previous index hip fracture. We also excluded patients with index fractures that occurred before January 1, 2014, the start of the study period. This gave us a wash-in period of at least two years regarding previous hip fractures. For example, if a patient was admitted to a certified hospital with an S72.0 hip fracture diagnosis on January 1, 2014, and was rehospitalized on January 1, 2016, for the same diagnosis, only the first record would be considered. However, if the patient had an additional S72.0 diagnosis on January 1, 2012, then all of this patient's hospitalization records would be excluded because their index fracture occurred before the study period began. Furthermore, we excluded hip fractures that occurred within one year of hospitalization for a fracture at another typical osteoporotic site. These latter fracture types were ICD-10 discharge diagnoses of the forearm (S52), humerus (S42), pelvis (S32.1, S32.3, S32.4, S32.5, S32.81, S32.83), or spine (S12.0–S12.2, S12.7, S12.9, S22.0, S22.1, S32.0). Excluding these follow-up fractures minimizes the possibility that the analyzed outcomes were impacted by a prior hip or other fragility fracture. For example, patients with fragility fractures are commonly readmitted to hospitals, e.g., due to injury to the fracture site, injury to another osteoporotic site, or other medical reasons [1]. The results of the analysis may be biased when patients whose health is still impacted by a recent fracture are included because survival probabilities and costs of follow-up treatment likely differ from those of index fractures of a new site [2]. We used larger exclusion windows for hip fractures following hospitalization for a previous hip fracture than for other fracture types because we considered outcomes at the same site to be more likely associated than outcomes at different sites. Additionally, we excluded patients who were already hospitalized with another diagnosis or in SR at the time of the index fracture (fewer than 3% of patients with surgically treated hip fractures across all orthogeriatric care models). Finally, we excluded patients who were transferred to SR within one day of hip fracture fixation, which typically takes one day.

Details on the statistical analysis of survival and costs

For the survival analyses, observations were left-truncated at the date of surgery and right-censored at a maximum follow-up of up to 180 days from index admission. For later deaths, the association with the hospital treatment of the index fracture was considered to be too weak to be of relevance based on previous research [3]. Nonparametric 180-day survival probabilities were computed with the Kaplan-Meier estimator stratified by the orthogeriatric care model (i.e., not assuming proportional hazards). Covariate-adjusted death hazard ratios

were estimated in Cox proportional hazards models. The proportional hazards assumption was checked based on Schoenfeld residual plots and tests. To accommodate non-proportional hazards, we fitted separate models for different follow-up time intervals. The time intervals were chosen by combining knowledge from the shape of the Schoenfeld residual plots and the treatment stages, like time of transfers in hospital networks or completion of EGR.

As healthcare costs, we conducted a cost-of-illness study where we considered the sum of all direct healthcare costs reimbursed for the inpatient index hospital, or the first SR stay that followed ("costs in hospital and SR") and corresponding LOS. All costs during consecutive stays since the admission due to an index hip fracture were summed up, including stays after transfers to external hospitals. The corresponding costs could be directly inferred from the claims data, so no approximation was necessary. To obtain complete observations of hospital and SR stays and to circumvent bias in empirical averages [4], LOS and costs were analyzed with an extended follow-up period of 365 days from the index admission. This implies that discharges from hospital or SR after 180 days were also considered, resulting in no censoring of the included patients' hospital or SR stays. Covariate-adjusted cost and LOS ratios were estimated in gamma regression models with a logarithmic link function. To reduce the influence of extreme outliers in the cost and LOS regression models, all costs and LOS were truncated at the 99% quantile of each orthogeriatric care model. All costs were adjusted for inflation using factors provided by the Organization for Economic Cooperation and Development (OECD) [5] with the most recent reference year 2018.

Supplementary tables and figures

Table S1: Cross table of orthogeriatric care model characteristics by the patients' index hospitals.

Integration of the geriatrician into the surgical ward	Frequency of geriatric visits to patients	Hospital network structure	Number of hospitals[1]	Orthogeriatric care model
GCS	twice a week	Single-site institution	26	low-frequency GCS
GCS	> twice a week	Single-site institution	46	high-frequency GCS
GCS	twice a week	Multisite cooperation	41	hospital network
GCS	> twice a week	Multisite cooperation	1	hospital network
ICM	> twice a week	Single-site institution	7	ICM
ICM	twice a week	Multisite cooperation	1	hospital network ICM
ICM	> twice a week	Multisite cooperation	2	hospital network ICM

[1]Number of index hospitals in the study population. GCS: geriatric consulting service, in which the geriatrician consults with the surgical ward. ICM: integrated care model, in which the geriatrician is a permanent member of the surgical ward.

In addition to the orthogeriatric care models analyzed in the main manuscript, three hospitals were identified that operate similarly to the hospital network model but use ICM instead of GCS in the surgical ward. However, they were excluded due to their low representation and incompatibility with the other models for a combined analysis. Specifically, patients in hospital networks are usually transferred for specialized geriatric treatment. In contrast, the ICM model aims to provide this treatment in the surgical ward, thereby reducing the need for transfer to network hospitals. Therefore, including these hospitals in the analysis would increase heterogeneity and standard errors in the statistical analyses (*Table S8*).

Table S2: Definition of hospital characteristics. EGR: early complex geriatric rehabilitation therapy.

Hospital volume	Definition	Population	Summarized by
Yearly treated hip fractures (surgical volume)	Number of treated fractures of the hip	All AOK insured patients, weighted by federal-state-level inverse insurance coverage proportions	Hospital and year
Yearly EGR (geriatric volume)	Number of reimbursed OPS8-550 treatments	All AOK insured patients, weighted by federal-state-level inverse insurance coverage proportions	Hospital and year

Table S3: Proportions of surgical treatment by the index hospitals' orthogeriatric care model.

**Characteristic**	**low-frequency GCS** 1595 patients[1]	**high-frequency GCS** 3698 patients[1]	**hospital network** 1974 patients[1]	**ICM** 490 patients[1]
Surgery	1,479 (93%)	3,451 (93%)	1,832 (93%)	457 (93%)
In-hospital deaths without surgery	14 (0.9%)	34 (0.9%)	24 (1.2%)	7 (1.4%)

[1]n (%). GCS: geriatric consulting service. ICM: integrated care model.

Table S4: Hospital counts and characteristics by orthogeriatric care model, summarized by hospital (with one observation for each hospital).

**Year**	**low-frequency GCS[1]**	**high-frequency GCS[1]**	**hospital network[1]**	**ICM[1]**
**Number of hospitals**				
2014	-	5	2	-
2015	9	19	8	1
2016	14	30	16	4
2017	19	36	33	4
2018	25	45	52	7
**Volume of treated hip fractures in the hospitals where patients underwent surgery**				
2014	-	297 (113.5)	388 (59.1)	-
2015	311 (179.0)	327 (141.8)	351 (142.7)	523 (-)
2016	308 (165.0)	376 (207.7)	359 (146.2)	381 (247.2)
2017	385 (200.3)	388 (198.2)	342 (138.6)	364 (216.2)
2018	395 (206.4)	554 (901.7)	418 (277.6)	345 (198.3)
**Volume of EGR in the hospitals where patients received EGR**				
2014	-	266 (182.0)	548 (104.6)	-
2015	409 (540.5)	345 (181.4)	667 (284.6)	287 (61.2)
2016	302 (289.2)	399 (260.4)	590 (218.9)	269 (121.0)
2017	460 (377.6)	365 (229.1)	488 (226.9)	183 (132.6)
2018	321 (262.2)	387 (448.2)	540 (454.6)	185 (151.7)

[1] Total count or mean (SD) of volumes per hospital. GCS: geriatric consulting service ICM: integrated care model. EGR: early complex geriatric rehabilitation therapy.

Table S5: Costs, length of stay, and adverse events in patient subgroups.

Characteristic	low-frequency GCS[1]	high-frequency GCS[1]	hospital network[1]	ICM[1]
Costs in all patients (thsnd. €)				
in hospital	10.9 (7.5, 12.3)	11.3 (7.7, 12.4)	11.1 (7.6, 13.3)	9.3 (7.5, 12.8)
in SR	3.79 (2.24, 4.17)	3.98 (3.02, 4.71)	3.81 (2.70, 4.82)	4.03 (2.54, 4.39)
in hospital and SR	11.5 (8.5, 13.3)	12.1 (9.1, 14.5)	11.8 (9.0, 13.8)	10.9 (8.3, 14.5)
Costs in patients discharged alive (thsnd. €)				
in hospital	10.9 (7.5, 12.3)	11.4 (7.8, 12.4)	11.2 (7.6, 13.2)	9.3 (7.5, 12.8)
in SR	3.89 (2.37, 4.19)	4.04 (3.35, 4.74)	3.81 (2.82, 4.85)	4.10 (2.54, 4.39)
in hospital and SR	12.2 (10.3, 15.6)	15.0 (11.7, 16.8)	12.6 (10.4, 15.8)	12.9 (10.4, 16.2)
Costs in patients with EGR (thsnd. €)				
in hospital	12.1 (11.4, 13.0)	12.1 (11.3, 12.9)	12.7 (11.4, 14.0)	12.1 (8.5, 13.4)
in SR	3.73 (2.33, 4.12)	4.04 (3.44, 4.74)	3.83 (2.99, 4.33)	4.15 (2.43, 4.39)
in hospital and SR	12.4 (11.7, 14.5)	12.9 (11.7, 15.9)	13.0 (11.8, 14.9)	12.3 (8.9, 14.8)
LOS in all patients (days)				
in hospital	20 (12, 26)	18 (13, 24)	23 (12, 31)	17 (13, 23)
in SR	21 (18, 23)	20 (19, 26)	20 (18, 25)	20 (19, 25)
LOS in hospital and SR	24 (14, 35)	26 (16, 37)	28 (16, 36)	23 (17, 35)
LOS in patients discharged alive (days)				
in hospital	20 (13, 26)	18 (14, 24)	24 (13, 31)	18 (14, 23)
in SR	21 (19, 23)	21 (20, 27)	20 (18, 25)	20 (19, 26)
in hospital and SR	39 (33, 47)	39 (35, 46)	39 (32, 46)	36 (32, 44)
LOS in patients with EGR (days)				
in hospital	25 (21, 30)	21 (17, 27)	29 (25, 34)	20 (17, 26)
in SR	20 (18, 23)	21 (19, 26)	20 (19, 22)	20 (19, 22)
in hospital and SR	28 (22, 39)	29 (21, 40)	31 (27, 39)	24 (18, 35)
Adverse events				
In-hospital death	140 (9.5%)	286 (8.3%)	169 (9.2%)	33 (7.2%)
Readmission within 180 days of discharge*	578 (43%)	1,348 (43%)	621 (37%)	161 (38%)
Death within 21 days of SR start†	1 (0.2%)	13 (1.0%)	1 (0.2%)	1 (0.7%)
Transfer back to hospital from SR†	30 (7.1%)	90 (7.2%)	29 (7.0%)	6 (4.0%)

[1]n (%) or median (1st quartile, 3rd quartile). Percentages are computed in all patients or in subgroups of patients discharged alive from hospital (*), or patients who started SR (†). thsnd: thousand. LOS: length of stay. GCS: geriatric consulting service. ICM: integrated care model. EGR: early complex geriatric rehabilitation therapy.

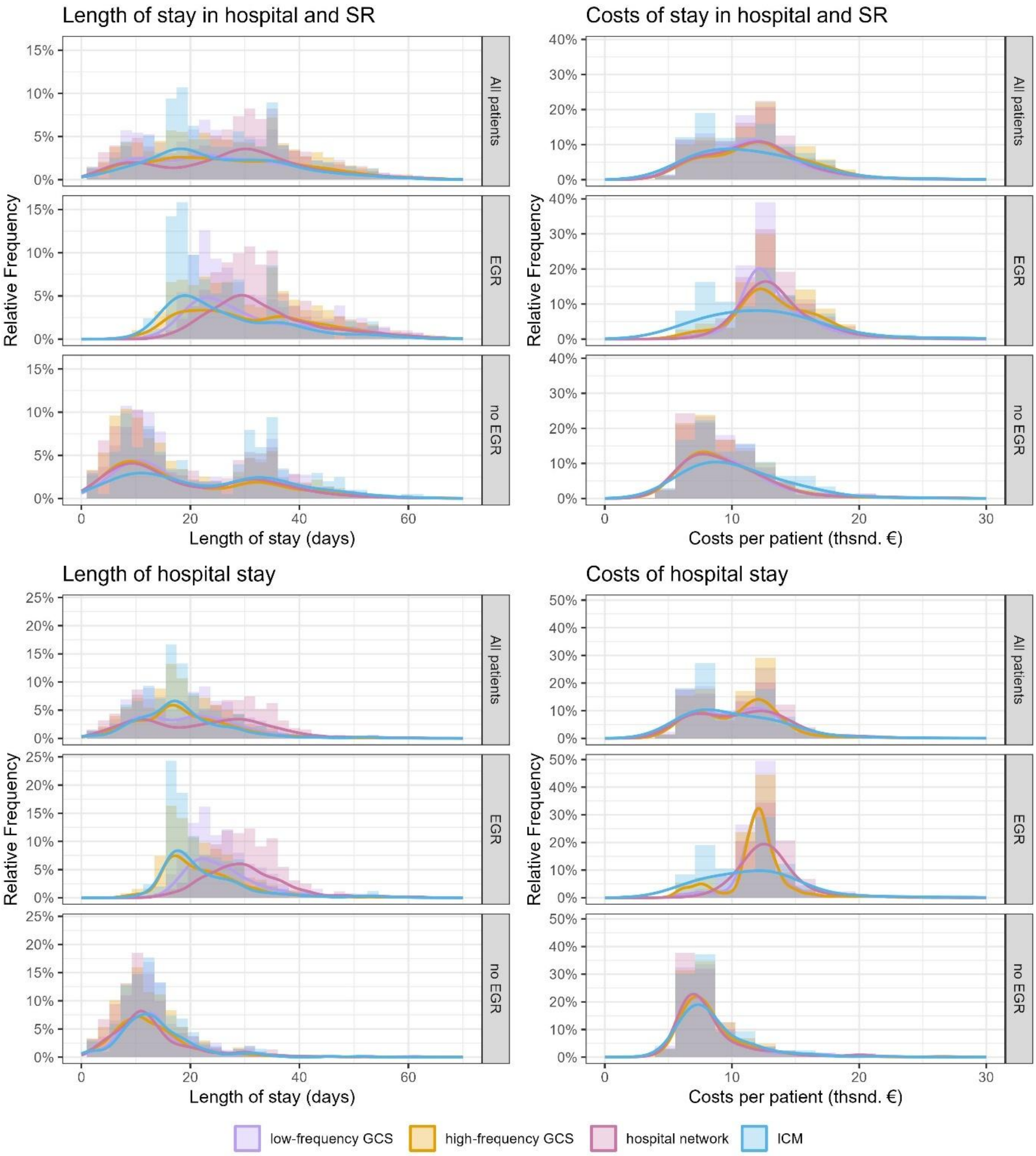

Figure S1: Distributions (histograms with kernel density estimates) of total time and inpatient costs in the hospitals and subacute rehabilitation (SR) facilities by type of orthogeriatric care model. The distributions are presented for all patients and subsets of patients who received or did not receive early geriatric rehabilitation therapy (EGR vs. no EGR). thsnd: thousand. GCS: geriatric consulting service. ICM: integrated care model.

Table S6: Cost ratio and death hazard ratio of low visit-frequency geriatric consult service versus orthogeriatric care models with more intensive geriatric involvement estimated in regression models with different covariate adjustments.

	Adjusted for			Low-frequency GCS (reference) vs.		
**Time**	**Sex, age**	**Health status**	**Regional factors**	**high-frequency GCS**	**hospital network**	**ICM**
				**Hazard ratio of death (95% CI)**		
(0,30] days	true	true	true	0.88 (0.75,1.02)	0.92 (0.74,1.15)	0.72 (0.60,0.87)
(0,30] days	true	true	false	0.86 (0.74,1.01)	0.91 (0.73,1.14)	0.74 (0.62,0.88)
(0,30] days	true	false	true	0.87 (0.74,1.03)	0.91 (0.73,1.15)	0.64 (0.53,0.79)
(0,30] days	true	false	false	0.85 (0.72,1.01)	0.91 (0.72,1.13)	0.66 (0.54,0.81)
(0,30] days	false	true	true	0.90 (0.77,1.04)	0.94 (0.76,1.18)	0.71 (0.60,0.85)
(0,30] days	false	true	false	0.87 (0.75,1.02)	0.93 (0.75,1.16)	0.74 (0.63,0.88)
(0,30] days	false	false	true	0.90 (0.76,1.06)	0.94 (0.74,1.18)	0.64 (0.53,0.77)
(0,30] days	false	false	false	0.87 (0.73,1.02)	0.92 (0.73,1.16)	0.67 (0.54,0.82)
(30,180] days	true	true	true	0.89 (0.77,1.01)	0.80 (0.67,0.94)	0.99 (0.80,1.23)
(30,180] days	true	true	false	0.88 (0.78,0.98)	0.79 (0.67,0.94)	1.01 (0.81,1.26)
(30,180] days	true	false	true	0.89 (0.76,1.05)	0.80 (0.67,0.96)	0.90 (0.73,1.12)
(30,180] days	true	false	false	0.88 (0.77,1.01)	0.80 (0.67,0.95)	0.91 (0.73,1.15)
(30,180] days	false	true	true	0.89 (0.77,1.03)	0.81 (0.68,0.96)	0.98 (0.79,1.21)
(30,180] days	false	true	false	0.88 (0.78,0.99)	0.80 (0.67,0.95)	1.00 (0.80,1.26)
(30,180] days	false	false	true	0.91 (0.77,1.08)	0.81 (0.67,0.98)	0.89 (0.72,1.11)
(30,180] days	false	false	false	0.89 (0.78,1.02)	0.80 (0.67,0.96)	0.92 (0.71,1.17)
				**Ratio of mean costs (95% CI)**		
hospital & SR	true	true	true	1.00 (0.98,1.03)	0.99 (0.96,1.02)	1.00 (0.95,1.05)
hospital & SR	true	true	false	1.02 (0.99,1.05)	1.00 (0.97,1.03)	0.98 (0.94,1.03)
hospital & SR	true	false	true	1.00 (0.98,1.03)	0.99 (0.96,1.02)	1.01 (0.97,1.06)
hospital & SR	true	false	false	1.02 (0.99,1.05)	1.00 (0.97,1.03)	1.00 (0.95,1.05)
hospital & SR	false	true	true	1.00 (0.98,1.03)	0.99 (0.96,1.02)	1.00 (0.95,1.04)
hospital & SR	false	true	false	1.02 (0.99,1.05)	1.00 (0.97,1.03)	0.98 (0.93,1.03)
hospital & SR	false	false	true	1.00 (0.98,1.03)	0.99 (0.96,1.02)	1.01 (0.96,1.06)
hospital & SR	false	false	false	1.02 (0.99,1.05)	1.00 (0.97,1.03)	0.99 (0.95,1.04)

Health status: medication-based comorbidity score, care level. Regional factors: German Index of Socioeconomic Deprivation. CI: confidence interval. GCS: geriatric consulting service. ICM: integrated care model. SR: subacute rehabilitation.

Table S7: Hazard ratios of death comparing different orthogeriatric care models in regression models that estimate the time of index admission onwards (without left truncation) and include non-operated hip fractures.

Pairwise comparisons	Hazard ratio[1] (0,30] days	Hazard ratio[1] (30,180] days
**Reference: low-frequency GCS**		
high-frequency GCS	0.95 (0.78,1.15)	0.90 (0.76,1.07)
hospital network	0.98 (0.75,1.29)	0.81 (0.66,1.004)
ICM	0.95 (0.68,1.33)	1.02 (0.80,1.30)
**Reference: high-frequency GCS**		
hospital network	1.04 (0.80,1.35)	0.90 (0.74,1.10)
ICM	1.00 (0.71,1.42)	1.13 (0.88,1.46)
**Reference: hospital network**		
ICM	0.97 (0.66,1.40)	1.26 (0.96,1.64)

[1]adjusted for age, sex, medication-based comorbidity score, care level, socioeconomic deprivation, within-hospital dependencies, and multiple testing with 95% confidence intervals. GCS: geriatric consulting service. ICM: integrated care model.

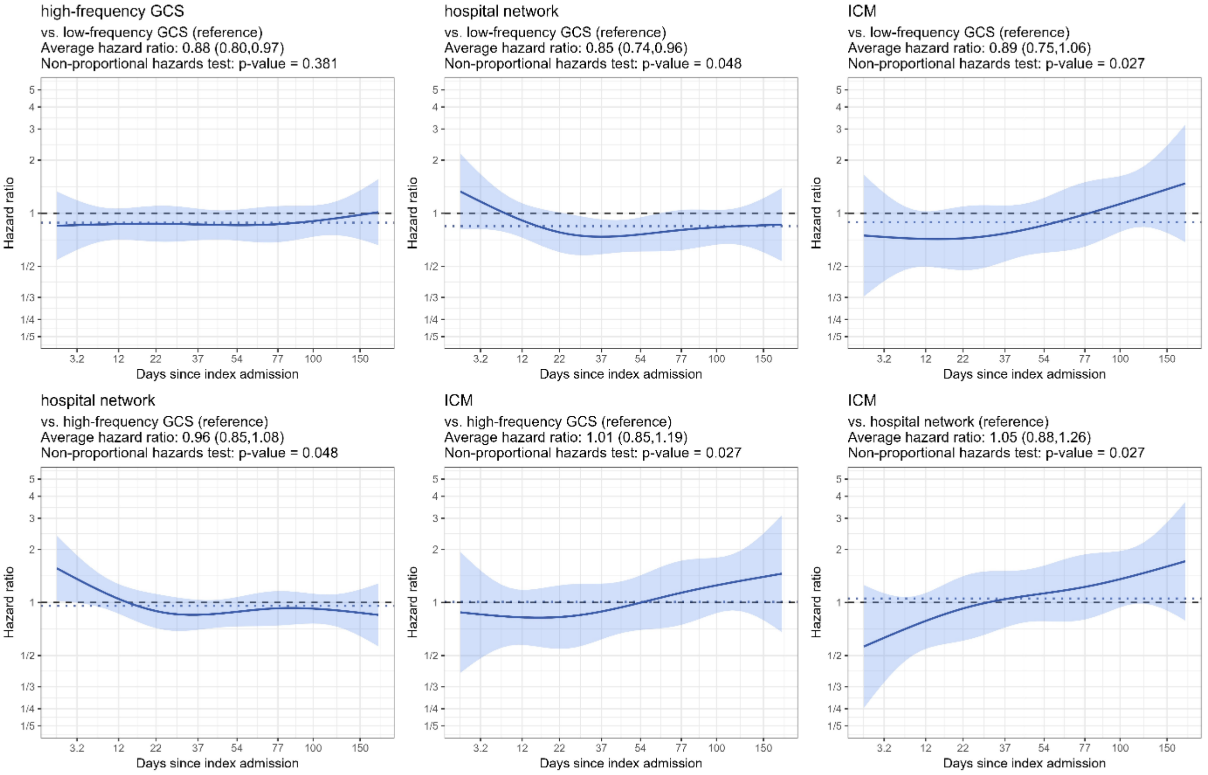

Figure S2: Schoenfeld tests and smoothed time-dependent hazard ratios of death comparing different orthogeriatric care models while adjusting for age, sex, medication-based comorbidity score, care level, socioeconomic deprivation, and within-hospital dependencies. GCS: geriatric consulting service. ICM: integrated care model.

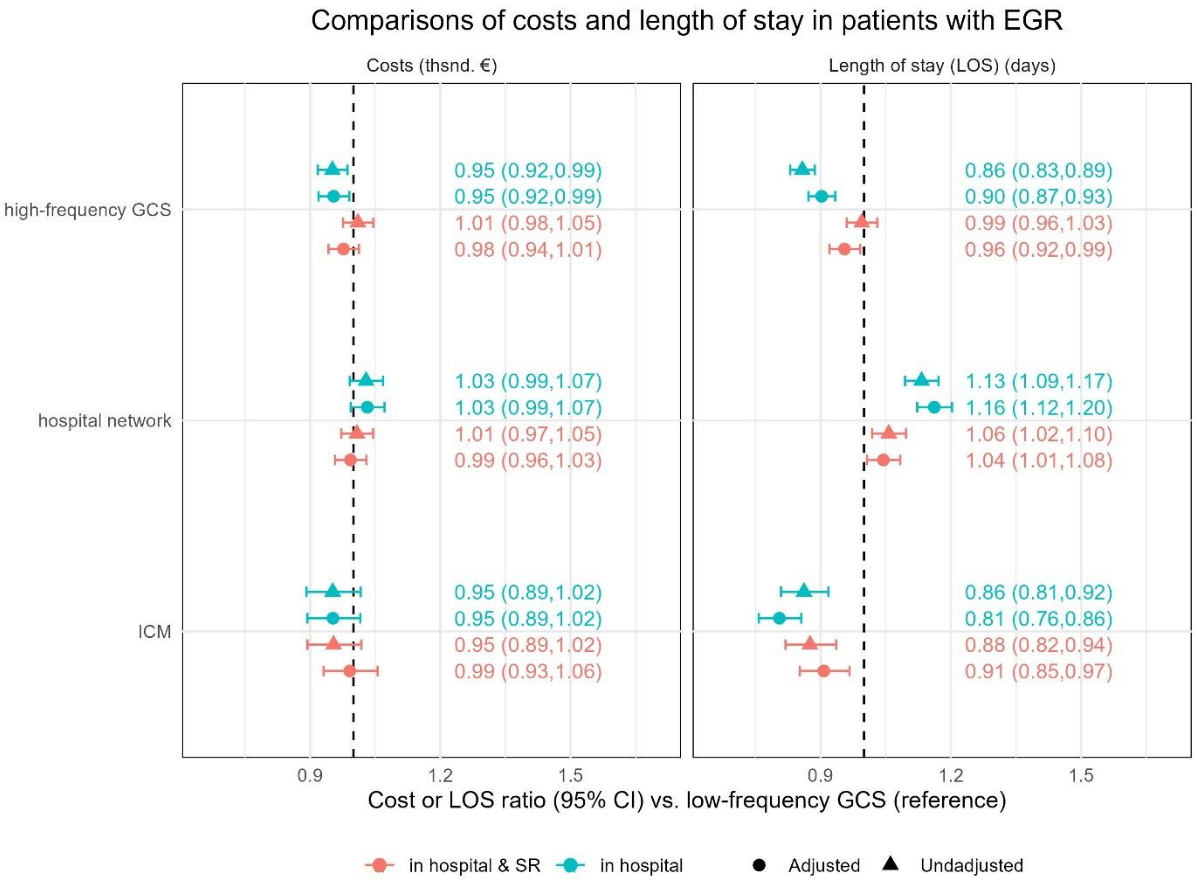

Figure S3: Cost ratio and length of stay (LOS) ratio of low visit-frequency geriatric consult service (GCS) versus orthogeriatric care models with more intensive geriatric involvement in patients with early geriatric rehabilitation therapy (EGR). Results are presented for unadjusted regression models and models adjusted for age, sex, medication-based comorbidity score, care level, socioeconomic deprivation, and within-hospital dependencies. ICM: integrated care model. SR: subacute rehabilitation.

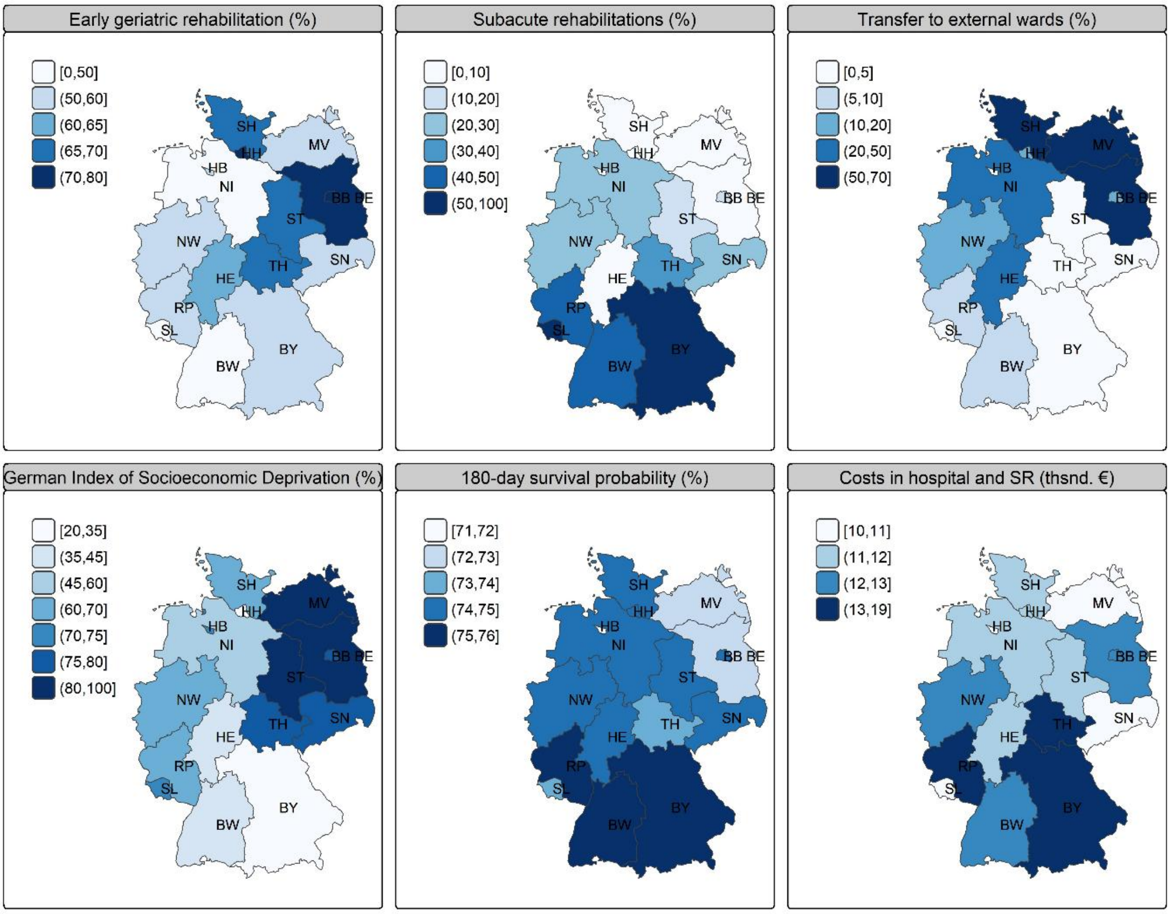

Figure S4: Distribution of rehabilitation and transfer characteristic and health and health-economic outcomes (survival, costs) in the study population and state-level socioeconomic deprivation. SR: subacute rehabilitation.

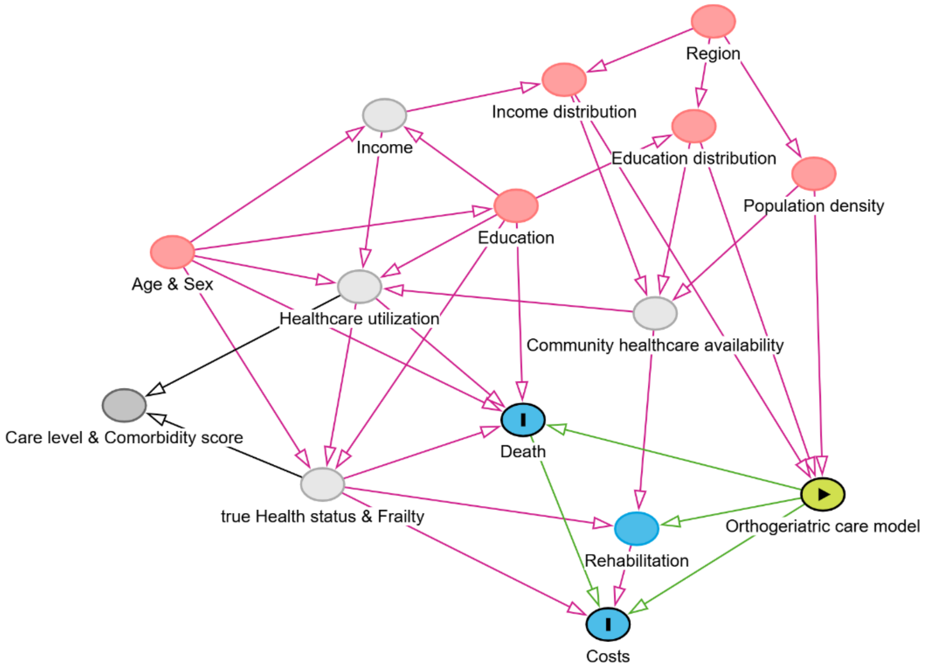

Figure S5: Directed acyclic graph displaying potential relationships between the orthogeriatric care model and the hazard of death or inpatient costs. The exposure is shown as green rectangle with a right-pointing triangle; outcomes and their ancestors as blue rectangles with vertical notches for the outcomes. Unobserved variables appear as white ovals. Edges are colored by role: green lines indicate causal paths; pink lines indicate biasing paths where present.

Table S8: Baseline characteristics by the hospitals' orthogeriatric care model, including ICM hospitals with multisite cooperation structures (hospital network ICM).

**Characteristic**	**low-frequency GCS**[1]	**high-frequency GCS**[1]	**hospital network**[1]	**ICM**[1]	**hospital network ICM**[1]
	1479 patients	3451 patients	1832 patients	457 patients	211 patients
	26 hospitals	46 hospitals	42 hospitals	7 hospitals	3 hospitals
Age at admission (years)	87 (84, 91)	87 (84, 91)	87 (83, 91)	87 (84, 91)	87 (84, 92)
Sex female	1,136 (77%)	2,606 (76%)	1,393 (76%)	376 (82%)	160 (76%)
Care level					
None or 1	485 (33%)	1,185 (34%)	601 (33%)	197 (43%)	86 (41%)
2-3	648 (44%)	1,416 (41%)	832 (45%)	182 (40%)	81 (38%)
4-5	346 (23%)	850 (25%)	399 (22%)	78 (17%)	44 (21%)
Resident of a nursing home	363 (25%)	844 (24%)	485 (26%)	97 (21%)	55 (26%)
Medication-based comorbidity score	4 (3, 5)	4 (3, 5)	4 (3, 6)	4 (2, 5)	4 (3, 6)
Inpatient costs in the previous year					
No costs in previous year	839 (57%)	1,966 (57%)	1,058 (58%)	281 (61%)	121 (57%)
<5 thsnd. €	336 (23%)	868 (25%)	445 (24%)	105 (23%)	54 (26%)
≥5 thsnd. €	304 (21%)	617 (18%)	329 (18%)	71 (16%)	36 (17%)
Surgery within 1 day of admission	1,275 (86%)	2,902 (84%)	1,549 (85%)	407 (89%)	180 (85%)
Year of index admission					
2014	0 (0%)	65 (1.9%)	58 (3.2%)	0 (0%)	0 (0%)
2015	205 (14%)	399 (12%)	88 (4.8%)	32 (7.0%)	13 (6.2%)
2016	285 (19%)	692 (20%)	224 (12%)	105 (23%)	40 (19%)
2017	435 (29%)	994 (29%)	606 (33%)	138 (30%)	71 (34%)
2018	554 (37%)	1,301 (38%)	856 (47%)	182 (40%)	87 (41%)
Hospital volume where patients underwent surgery:					
Yearly treated hip fractures	419 (308, 503)	415 (302, 565)	406 (309, 500)	523 (306, 650)	222 (181, 566)
Yearly EGR	244 (167, 356)	285 (206, 504)	0 (0, 186)	372 (72, 395)	0 (0, 0)
Hospital volume where patients received EGR:					
Yearly treated fragility fractures	397 (278, 531)	406 (296, 544)	337 (261, 440)	646 (306, 650)	475 (204, 498)
Yearly EGR	338 (222, 416)	303 (212, 511)	501 (337, 693)	372 (112, 395)	786 (212, 831)
Socioeconomic factors in patients' residential state:					
German Index of Socioeconomic Deprivation (%)	67 (38, 68)	36 (35, 65)	46 (38, 67)	100 (36, 100)	70 (67, 70)

[1]n (%) or median (1st quartile, 3rd quartile). thsnd: thousand. EGR: early complex geriatric rehabilitation therapy. GCS: geriatric consulting service. ICM: integrated care model.

References

1. Unseld T, Rapp K, Becker C, Konnopka C, König H-H, Jaensch A, et al. Orthogeriatric co-management and risk of rehospitalization in older patients with osteoporotic fractures: a retrospective cohort study from Germany. BMC Geriatr. 2025;25:566. https://doi.org/10.1186/s12877-025-06172-5.

2. Boockvar KS, Halm EA, Litke A, Silberzweig SB, McLaughlin M, Penrod JD, et al. Hospital Readmissions After Hospital Discharge for Hip Fracture: Surgical and Nonsurgical Causes and Effect on Outcomes. J Am Geriatr Soc. 2003;51:399–403. https://doi.org/10.1046/j.1532-5415.2003.51115.x.

3. Rapp K, Roigk P, Becker C, Todd C, Rehm M, Rothenbacher D, et al. Association of two geriatric treatment systems with anti-osteoporotic drug treatment and second hip fracture in patients with an index hip fracture: retrospective cohort study. BMC Geriatr. 2024;24:395. https://doi.org/10.1186/s12877-024-04989-0.

4. Keogh RH, Diaz-Ordaz K, Jewell NP, Semple MG, de Wreede LC, Putter H, et al. Estimating distribution of length of stay in a multi-state model conditional on the pathway, with an application to patients hospitalised with Covid-19. Lifetime Data Anal. 2023;29:288–317. https://doi.org/10.1007/s10985-022-09586-0.

5. Organisation for Economic Co-operation and Development. Consumer Prices – OECD Data Explorer. 2025.
